# Mitochondrial tRNA^Ser(AGY)^ 12234A > G Mutation May Contribute to the Clinical Expression of Hypertrophic Cardiomyopathy‐Associated tRNA^Ile^ 4263A > G Mutation

**DOI:** 10.1155/humu/6788682

**Published:** 2026-08-12

**Authors:** Mengting Liu, Qinxian Guo, Shunrong Zhang, Daojun Yu, Yu Ding

**Affiliations:** ^1^ The Fourth School of Clinical Medicine, Zhejiang Chinese Medical University, Hangzhou First People′s Hospital, Hangzhou, Zhejiang, China, zcmu.edu.cn; ^2^ Department of Clinical Laboratory, Affiliated Hangzhou First People′s Hospital, School of Medicine, Westlake University, Hangzhou, Zhejiang, China, westlake.edu.cn; ^3^ Department of Geriatrics, Affiliated Hangzhou First People′s Hospital, School of Medicine, Westlake University, Hangzhou, Zhejiang, China, westlake.edu.cn

**Keywords:** hypertrophic cardiomyopathy, m.12234A > G, m.4263A > G, mitochondrial tRNA mutations, synergy

## Abstract

Mutations in mitochondrial tRNA (mt‐tRNA) are found to be associated with hypertrophic cardiomyopathy (HCM), but their molecular mechanisms remain largely undetermined. In this study, we investigated the contribution of a novel HCM‐related mt‐tRNA^Ser(AGY)^ 12234A > G mutation to the phenotypic expression of the mt‐tRNA^Ile^ 4263A > G mutation in two genetically unrelated Han Chinese pedigrees. Strikingly, the penetrance and expressivity of one pedigree (HCM2) with both m.4263A > G and m.12234A > G mutations are much higher than another pedigree (HCM1) with only m.4263A > G mutation. By molecular level, the homoplasmic m.4263A > G mutation is located at the processing site for the tRNA^Ile^ 5 ^′^‐end precursor, disrupting a conserved Watson–Crick base pairing (1A–69T) which is believed to cause mitochondrial dysfunction. Moreover, the heteroplasmic m.12234A > G mutation occurs at an extremely conserved nucleotide in the anticodon stem of tRNA^Ser(AGY)^, a position which is critical for tRNA structure and function. Using trans‐mitochondrial cell models, we demonstrated that cybrids with both mt‐tRNA mutations exhibited more severe mitochondrial dysfunctions than cybrids with only the m.4263A > G mutation. Furthermore, a marked decrease in mt‐RNA transcripts was observed in cells harboring both m.4263A > G and m.12234A > G mutations. Taken together, our study indicated that the m.12234A > G mutation acted in synergy with the m.4263A > G mutation, triggering mitochondrial dysfunctions and contributing to a high penetrance of HCM in a pedigree harboring both mtDNA mutations.

## 1. Introduction

Hypertrophic cardiomyopathy (HCM) is a heritable cardiovascular disorder, affecting approximately 1 in 500 individuals [1]. HCM progresses through a compensatory response relying on changes in gene expression, bioenergetics, and cellular morphology of cardiomyocytes. It is characterized by left ventricular hypertrophy and diastolic dysfunction and increases the risk of arrhythmias and heart failure [2]. In particular, HCM is the most common cause of sudden cardiac death in individuals younger than 35 years old [3]. It has been well accepted that HCM can be caused by multiple factors including environmental toxins, neuromuscular disorders, and genetic factors [4]. Among genetic risk factors, mutations in sarcomere and sarcomere‐linked protein genes account for the greatest contributors to HCM cases [5, 6]. For instance, experimental studies showed that mutations in Myosin Heavy Chain 7 (*MYH7*) and cardiac myosin‐binding protein C (*MYBPC*) explained 60%–70% of observed clinical cases [7]. Nevertheless, these studies are mainly focused on nuclear genes. The roles of mitochondrial genetic variants in HCM remain poorly understood.

The heart is a highly energetically demanding organ that sources 90% of the ATP from mitochondrial oxidative phosphorylation (OXPHOS) [8]. The human mitochondrion is a semiautonomous organelle and contains its own genome (mitochondrial DNA [mtDNA]), encoding 13 subunits for Complexes I~V, as well as two rRNAs and 22 tRNAs necessary for mitochondrial protein translation [9]. Due to the lack of histone protection and a poor DNA repair system, mtDNA has a higher mutation rate than nuclear DNA (nDNA) [10]. Early studies demonstrated that maternal transmission of cardiomyopathy had been implicated in several pedigrees [11, 12], highlighting the importance of mtDNA mutations to this disease. More recently, some case studies have shown that mitochondrial tRNAs (mt‐tRNAs) were the hot spots for pathogenic mutations associated with HCM; for instance, tRNA^Ile^ 4277 T > C, 4295A > G, and 4300A > G [13–15], tRNA^Leu(UUR)^ 3303C > T [16], and tRNA^Gly^ 9997 T > C [17] were implicated to affect mtDNA translation, causing defects in protein synthesis and decreasing OXPHOS functions. However, due to the complex etiology of mitochondrial cardiomyopathy, the roles of mt‐tRNA mutations in HCM are still largely unknown.

To explore whether mitochondrial genetic defects contribute to the pathogenesis of HCM, we recently initiated a systematic mutational screening of mt‐tRNA in a cohort of 421 patients with HCM and 589 control subjects from Hangzhou City in Zhejiang Province, China. This analysis led us to identify two Han Chinese families with maternally inherited HCM carrying a homoplasmic tRNA^Ile^ 4263A > G mutation. Intriguingly, we identified a novel heteroplasmic tRNA^Ser(AGY)^ 12234A > G mutation, together with a tRNA^Ile^ 4263A > G mutation, in one Chinese pedigree (HCM2) which exhibited much higher penetrance and expressivity of cardiomyopathy than another pedigree (HCM1) with only m.4263A > G mutation. Notably, the m.12234A > G mutation occurred at a conventional Position 42 in the anticodon stem of mt‐tRNA^Ser(AGY)^, which was conserved from different species. Importantly, the well‐known m.15927G > A mutation, which was also located at the same position of tRNA^Thr^, had been implicated in causing mitochondrial dysfunctions that were involved in coronary heart disease pathogenesis [18]. Therefore, we hypothesized that the m.12234A > G mutation, which was similar to the m.15927G > A mutation, could also impair mitochondrial function that is involved in HCM expression. To investigate the synergistic effect of the m.12234A > G to m.4263A > G mutation, we established cytoplasmic hybrid (cybrid) cells derived from three HCM patients with both m.12234A > G and m.4263A > G mutations, three patients with only the m.4263A > G mutation, and three healthy individuals without these mtDNA mutations. These cell lines were then assayed for the effects of mt‐tRNA mutations on mitochondrial functions.

## 2. Materials and Methods

### 2.1. Pedigree Information

**Figure 1:**
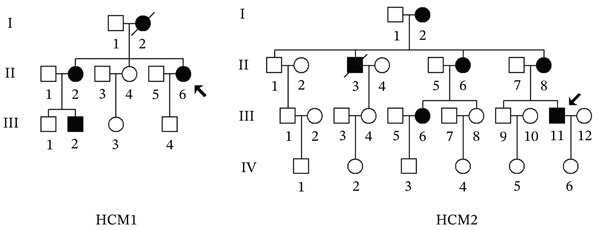
Two Han Chinese pedigrees with maternally transmitted HCM; affected individuals are indicated by filled symbols. Arrows denote the probands.

As part of a genetic screening program for HCM‐associated mt‐tRNA mutations, two Han Chinese families (HCM1 and HCM2), as shown in Figure 1, were ascertained in Hangzhou First People′s Hospital. This study was conducted according to the Declaration of Helsinki. Informed consent and blood samples were obtained from all participants under the protocol approved by Hangzhou First People′s Hospital (Approval Number: KY‐20240408‐0123‐01). Furthermore, 589 healthy subjects, including 300 males and 289 females, aged from 44 to 60 years, with an average of 52 years, were recruited from the Physical Examination Center of Hangzhou First People′s Hospital as controls. All participants provided written informed consent.

### 2.2. Clinical Evaluations

We first administered a questionnaire to record the proband′s medical history and the abnormalities of other clinical disorders. The HCM subjects underwent a standard two‐dimensional transthoracic echocardiographic evaluation, which included the measurements of maximum left ventricular wall thickness (MLVWT), interventricular septum (IVS) thickness, left posterior wall (LPW) thickness, and ejection fraction (EF). Furthermore, standard 12‐lead electrocardiograms (ECGs) were obtained from the matrilineal relatives of these pedigrees during quiet respiration. Cardiac rhythm, PR interval, QRS duration, ST segment, and T‐wave were recorded as previously suggested [19].

The HCM was defined according to guidelines proposed by Joint Committee of American College of Cardiology/American Heart Association [20]: (1) A two‐dimensional echocardiography or cardiovascular magnetic resonance (CMR) shows a maximal end‐diastolic wall thickness of ≥ 15 mm (13–14 mm can be diagnostic when present in a family history or positive genetic testing), and (2) the hypertrophy is typically asymmetric in distribution: IVS/LPW ≥ 1.3.

### 2.3. Whole‐Exome Sequencing (WES) and Bioinformatics Analysis

Genomic DNA of HCM patients from two pedigrees (HCM1: II‐2, II‐6, and III‐2; HCM2: II‐6, II‐8, and III‐1) was isolated using a TaKaRa Blood Genome DNA Extraction Kit (TaKaRa). We first established a DNA library by using the SureSelect Library Prep Kit (Agilent). Subsequently, WES was performed using the Illumina NovaSeq 6000 platform (Illumina), according to a previous study [21]. The sequencing depth averaged a minimum of 200×. Raw sequencing reads were aligned to the GRCh37/hg19 reference genome using BWA‐MEM (v0.7.17). Variant calling was performed using GATK4 (v4.1.2). Variants were filtered using the following criteria: (1) minor allele frequency (MAF) < 0.01 in the 1000 Genomes Project, gnomAD, and an in‐house control database (*n* = 589); (2) read depth ≥ 20× and genotype quality ≥ 30; and (3) variant types: nonsynonymous, splice‐site (±2 bp), and frameshift indels.

Functional impact was predicted using SIFT, PolyPhen‐2, and MutationTaster. Variants predicted to be damaging by at least two tools were retained for further analysis. Known HCM‐associated nuclear genes, including *MYH7*, *MYBPC3*, *TNNI3*, *TNNT2*, *TPM1*, *ACTC1*, *MYL2*, *MYL3*, and *PLN*, were specifically examined for coverage and rare variants. All these genes had an average coverage > 150×, with > 98% of coding regions covered at ≥ 20×, ensuring reliable variant detection. The bioinformatics analysis pipeline followed a previously described method [22].

### 2.4. Analysis of mtDNA Mutations

We used PCR–Sanger sequencing analysis to detect mtDNA mutations in matrilineal relatives in HCM1 and HCM2 pedigrees. PCR was performed using 24 primers, as described in our previous study [23]. The PCR products spanning the whole mitochondrial genomes were purified and sequenced. The data were compared with revised Cambridge reference sequences (rCRSs) to detect mutations (GenBank Accessible Number: NC_012920.1) [24].

The frequencies of mt‐tRNA^Ile^ 4263A > G and mt‐tRNA^Ser(AGY)^ 12234A > G mutations were further examined in 589 control subjects. The primers for amplification of the region spanning the mt‐tRNA^Ile^ gene were as follows: forward 5 ^′^‐TGG CTC CTT TAA CCT CTC CA‐3 ^′^; reverse: 5 ^′^‐AAG GAT TAT GGA TGC GGT TG‐3 ^′^; the specific primers used to amplify the region covering the mt‐tRNA^Ser(AGY)^ gene were as follows: forward: 5 ^′^‐TAT CAC TCT CCT ACT TAC AG‐3 ^′^; reverse: 5 ^′^‐AGA AGG TTA TAA TTC CTA CG‐3 ^′^. PCR products were further analyzed by Sanger sequencing, as described above.

### 2.5. Evaluations of mtDNA Pathogenic Mutations

We used the Clustal W program (https://www.genome.jp/tools-bin/clustalw) to determine the conservation index (CI) of each mtDNA mutation. A CI ≥ 75*%* was regarded as having functional importance [25]. Furthermore, the MITOMAP (www.mitomap.org/MITOMAP), mtDB (https://www.mtdb.igp.uu.se/), and mtSNP (http://mtsnp.tmig.or.jp) databases were used to evaluate the potential pathogenicity of mtDNA mutations. The mitochondrial haplogroups of two pedigrees were classified according to a previous study by Kong et al. [26].

### 2.6. Analysis of 8‐OHdG Concentrations in Plasma

The blood samples of each matrilineal relative (HCM1: II‐2, II‐6, and III‐2; HCM2: II‐6, II‐8, and III‐1), as well as three controls without these mutations (C1, C2, and C3), were centrifuged at 3000 g for 10 min at 4°C to collect plasma. The concentrations of plasma 8‐OHdG were determined via a specific ELISA kit (ab201734, Abcam). Absorbances at 450 nm were read in a microplate reader [27].

### 2.7. Quantification of mtDNA Copy Number

The peripheral blood mtDNA copy number in three patients with both m.4263A > G and m.12234A > G mutations (HCM2: II‐6, II‐8, and III‐11), three patients with only m.4263A > G mutation (HCM1: II‐2, II‐6, and III‐2), and three controls without these mtDNA mutations (C1, C2, and C3) were examined by quantitative real‐time PCR according to our previous study [28]. The primers for amplification of MT‐ND1 were as follows: forward: 5 ^′^‐AAC ATA CCC ATG GCC AAC CT‐3 ^′^; reverse: 5 ^′^‐AGC GAA GGG TTG TAG TAG CCC‐3 ^′^. The primers for amplification of the *β*‐globin gene were as follows: forward: 5 ^′^‐GAA GAG CCA AGG ACA GGT AC‐3 ^′^; reverse: 5 ^′^‐CAA CTT CAT CCA CGT TCA CC‐3 ^′^. It was calculated based on the following formula: mtDNA copy number per cell = 2 × (MT − ND1 copies/*β* − globin gene copies) [29].

### 2.8. Cell Lines and Cultured Conditions

Approximately 5 mL of venous blood was collected from three patients carrying both m.4263A > G and m.12234A > G mutations (HCM2: II‐6, II‐8, and III‐11), three subjects with only the m.4263A > G mutation (HCM1: II‐2, II‐6, and III‐2), and three controls (C1, C2, and C3) enrolled in this study. Trans‐mitochondrial cells were generated by fusing 143B *ρ* ^0^ cells (species: *Homo sapiens*; sex: female; tissue of origin: bone; official cell line name: 143B/206 rho 0) with platelets from the patients and controls, as described previously [30]. The *ρ* ^0^ cells were a gift from Dr. Hezhi Fang from Wenzhou Medical University in 2020 and were free of mycoplasma contamination. Cybrid cells were cultured in high‐glucose Dulbecco′s Modified Eagle′s Medium, supplied with 1% penicillin–streptomycin and 10% fetal bovine serum (FBS).

We next used PCR–Sanger sequencing to examine the presence of m.4263A > G and m.12234A > G mutations in cybrid cells. The primers for amplification of mt‐tRNA^Ile^ were as follows: forward: 5 ^′^‐TGG CTC CTT TAA CCT CTC CA‐3 ^′^; reverse: 5 ^′^‐AAG GAT TAT GGA TGC GGT TG‐3 ^′^; the primers for amplification of mt‐tRNA^Ser(AGY)^ were as follows: forward: 5 ^′^‐TAT CAC TCT CCT ACT TAC AG‐3 ^′^; reverse: 5 ^′^‐AGA AGG TTA TAA TTC CTA CG‐3 ^′^. After PCR and electrophoresis, the products were purified and sequenced, and the data were then compared with rCRS to detect the occurrence of m.4263A > G and m.12234A > G mutations in cybrids.

### 2.9. Analyses of Mitochondrial ATP Production

The ATP levels in nine cybrid cells were examined by CellTiter‐Glo Luminescent Cell Viability Assay Kit (Promega), as suggested previously [31].

### 2.10. Measurement of Mitochondrial Membrane Potential (MMP)

The MMP in nine cybrids was assayed by JC‐10 Assay Kit‐Microplate (Abcam) according to the manufacturer′s general recommendations, as detailed elsewhere [32].

### 2.11. ROS Analysis

The ROS production in various cell lines was determined by the 2 ^′^,7 ^′^‐Dichlorodihydrofluorescein‐Diacetate (DCFH‐DA) Kit (Beyotime), as detailed elsewhere [33].

### 2.12. Analysis of NAD^+^/NADH Ratio

The ratios of NAD^+^/NADH in nine cell lines were determined by NAD^+^/NADH Assay Kit (Abcam), according to the manufacturer′s instructions [34].

### 2.13. Determining the Calcium Ion Levels

The relative Ca^2+^ levels in nine cybrids were analyzed by Mitochondrial Calcium Assay Kit with Rhod‐2 AM (Abcam), according to the protocol generated by the manufacturer [35].

### 2.14. Lactate Measurement

The extracellular lactate levels in nine cybrids were analyzed using a fluorimetric‐based lactate assay kit (Amplite), according to the manufacturer′s instructions [36].

### 2.15. Analysis of mt‐RNA Transcription

To see whether the m.4263A > G and m.12234A > G mutations affected mt‐RNA transcription, we first isolated RNA from nine cybrid cells (~2 × 10^6^ cells) using TRIzol reagent (Thermo). Subsequently, the RNA was reverse‐transcribed and analyzed by qPCR, based on the protocol as described in our earlier study [37]. The expression levels of mt‐RNA transcripts were normalized to the endogenous reference gene GAPDH.

### 2.16. Statistical Analyses

All experiments were performed in triplicate. The data were expressed as mean ± standard deviation (SD). Statistical analyses were performed using SPSS 22.0 (IBM) software. Group comparisons were performed using the Kruskal–Wallis test followed by Dunn′s post hoc test with Bonferroni correction. Differences were considered significant at *p* < 0.05.

## 3. Results

### 3.1. Clinical Findings

**Figure 2:**
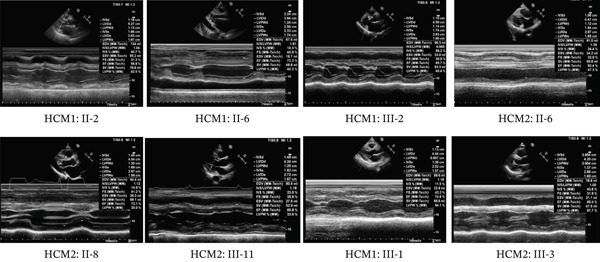
Clinical examination of matrilineal relatives who suffered from HCM using a two‐dimensional echocardiogram.

As shown in Figure 1, the proband (II‐6) of the HCM1 pedigree was a 65‐year‐old woman who lived in Hangzhou city of Zhejiang Province. She went to Hangzhou First People′s Hospital for treatment of HCM. ECHO result showed that the proband had 22.4 mm MLVWT and 12.4 mm LPW, which was an asymmetric hypertrophic distribution (IVS/LPW = 1.81). ECG showed that she had atrial fibrillation. Comprehensive family history interviewing indicated that family members (II‐2 and III‐2) were also affected with HCM (Figure 2).

HCM2 is a four‐generation Han Chinese pedigree; among 11 matrilineal relatives, six of them suffered from HCM at different ages at onset. Notably, the age at onset of HCM varied from 33 to 60 years, with an average of 48 years. Family history indicated that the proband′s uncle (II‐2) died from heart failure 2 years ago. The proband (III‐11) had an abnormal ECG (atrial fibrillation and pacing) at the age of 33 and was diagnosed with HCM. As shown in Figure 2 and Table 1, Subject III‐11′s MLVWT was 14.8 mm, which was much thicker than control subjects. The proband′s mother (II‐6) was diagnosed with HCM when she was 60. ECHO showed that her MLVWT and IVS were 15.6 mm. ECG revealed that she had atrial fibrillation. Furthermore, Subject II‐8 was diagnosed with HCM with MLVWT = 14.5 mm and IVS/LPW = 1.12.

**Table 1:** Clinical data for several matrilineal relatives in two pedigrees with HCM.

Subject	Gender	Age at test (year)	Age at onset (year)	BP (mmHg)	MLVWT (mm)	IVS thickness (mm)	LPW thickness (mm)	IVS thickness/LPW thickness	EF (%)	ECG rhythm	Abnormal Q‐wave	ST‐T change
HCM1: II‐2	Female	66	62	106/71	11.8	11.8	11.3	1.04	58.8	Sinus	+	+
HCM1: II‐6	Female	65	59	104/68	22.4	22.4	12.4	1.81	72.3	Atrial fibrillation	−	+
HCM1: III‐2	Male	42	41	118/67	11.4	11.0	11.4	0.97	65.7	Atrial fibrillation	+	+
HCM2: II‐6	Female	65	60	105/63	15.6	15.6	11.2	1.39	62.4	Atrial fibrillation	−	+
HCM2: II‐8	Female	68	51	136/72	14.5	14.5	13.0	1.12	72.1	Atrial fibrillation	+	+
HCM2: III‐11	Male	33	33	128/86	14.8	14.8	12.5	1.18	65.8	Atrial fibrillation and pacing	+	+
HCM1: III‐1	Male	35	/	124/83	11.3	11.3	9.57	1.18	74.6	Sinus	−	−
HCM2: III‐3	Male	32	/	131/85	9.54	9.54	9.54	1.0	60.4	Sinus	−	−

Abbreviations: BP, blood pressure; ECG, electrocardiography; EF, ejection fraction; IVS, interventricular septum; LPW, left posterior wall; MLVWT, maximum left ventricular wall thickness.

### 3.2. WES Analysis

To investigate whether nuclear gene mutations contributed to the pathogenesis of HCM in these pedigrees, WES was performed on the matrilineal relatives of two HCM pedigrees. After filtering against public databases (1000 Genomes Project, gnomAD) and an in‐house control database (*n* = 589) with a MAF threshold of < 0.01, no rare nonsynonymous, splice‐site, or loss‐of‐function mutations were identified in any known HCM‐associated nuclear genes, including *MYH7*, *MYBPC3*, *TNNI3*, *TNNT2*, *TPM1*, *ACTC1*, *MYL2*, *MYL3*, and *PLN*. The average sequencing coverage of these genes exceeded 150×, with > 98% of coding regions covered at ≥ 20×, ensuring reliable variant detection. These results suggested that nuclear gene mutations were unlikely to be the primary cause of HCM in these two pedigrees.

### 3.3. mtDNA Mutation Evaluations

**Figure 3:**
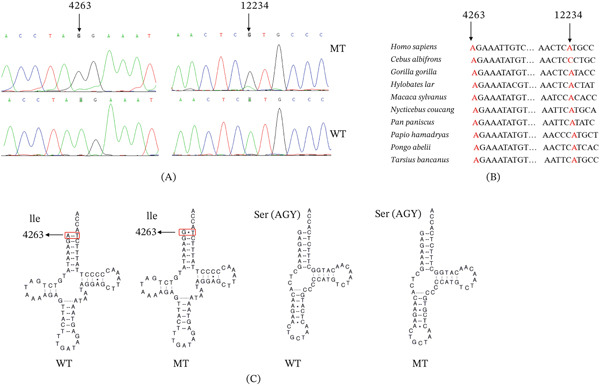
Molecular characterization of HCM‐related m.4263A > G and m.12234A > G mutations. (A) Identification of m.4263A > G and m.12234A > G mutations by Sanger sequencing. (B) Conservation assessments of the m.4263A > G and m.12234A > G mutations in different species. (C) Locations of m.4263A > G in tRNA^Ile^ and m.12234A > G mutation in tRNA^Ser(AGY)^. WT: wild type; MT: mutant.

Due to the maternally transmitted pattern, suggesting that mitochondria played a putative role in HCM progression, we next analyzed the mtDNA mutations in matrilineal relatives of two pedigrees (Table 2). Sequence analysis revealed the presence of 43 mutations, which belonged to East Asian mtDNA haplogroup D4j and D4, respectively [26]. Of these variants, there were 12 variants in the D‐loop, two variants in 12S rRNA and three variants in 16S rRNA, two mutations in tRNAs (tRNA^Ile^ and tRNA^Ser(AGY)^), while the remaining mutations were located at OXPHOS‐related genes. Furthermore, there were 10 missense mutations, including *ND1* 4048G > A (p.D248N), *ND2* 5178C > A (p.L237M), *A8* 8414C > T (p.L17F), *A6* 8701A > G (p.T59A) and 8860A > G (p.T112A), *ND3* 10398A > G (p.T112A), and *CytB* 14766C > T (p.T7I), 15326G > A (p.T194A), and 15884G > A (p.A380T). All these mutations were analyzed via phylogenetic conservation assessments in species including human, bovine [38], mouse [39], and *Xenopus laevis* [40]. It was found that except for the m.4263A > G and m.12234A > G mutations (Figure 3A,B), the others were not conserved, suggesting that they may not be involved in HCM pathogenesis.

**Table 2:** Mitochondrial DNA mutations identified in two pedigrees with HCM.

Gene	Position	Mutation	Amino acid change	Conservation (H/B/M/X)^a^	HCM1	HCM2	rCRS	Previously reported^b^
D‐loop	73	A to G			G	G	A	Yes
	146	T to C			C		T	Yes
	152	T to C				C	T	Yes
	310	T to CTC			CTC		T	Yes
	489	T to C				C	T	Yes
	524	Del C			Del C		C	Yes
	16051	A to G			G		A	Yes
	16111	C to T				T	C	Yes
	16189	T to C			C	C	T	Yes
	16223	C to T				T	C	Yes
	16519	T to C				C	T	Yes
	16569	T to C			C		T	Yes

12S rRNA	750	A to G		A/A/A/−	G	G	A	Yes
	1438	A to G		A/A/A/G	G	G	A	Yes

16S rRNA	2706	A to G		A/G/A/A	G	G	A	Yes
	3010	G to A		G/G/A/A	G		A	Yes
	3107	Del N			Del N	Del N	N	Yes

*ND1*	3970	C to T			T	T	C	Yes
	4048	G to A	Asp to Asn	D/N/Y/F		A	G	Yes

tRNA^Ile^	4263	A to G		A/A/A/A	G	G	A	Yes
*ND2*	5178	C to A	Leu to Met	L/T/T/T	A		C	Yes
	5301	A to G	Ile to Val	I/I/M/L		G	A	Yes
	5417	G to A				A	G	Yes

*CO1*	7028	C to T			T	T	C	Yes
*CO2*	8020	G to A			A		G	Yes
NC_7	8271‐79	Del 9‐bp			Del 9‐bp	Del 9‐bp	9‐bp	Yes
*A8*	8414	C to T	Leu to Phe	L/F/M/W	T	T	C	Yes

*A6*	8701	A to G	Thr to Ala	T/S/L/Q	G	G	A	Yes
	8860	A to G	Thr to Ala	T/A/A/T	G	G	A	Yes

*CO3*	9540	T to C			C		T	Yes
	9954	T to C				C	T	Yes
	9966	G to A				A	G	Yes

*ND3*	10398	A to G	Thr to Ala	T/T/T/A	G	G	A	Yes
	10400	C to T			T	T	C	Yes

*ND4*	10873	T to C			C		T	Yes
tRNA^Ser(AGY)^	12234	A to G		A/A/A/A		G	A	No
*ND6*	14668	C to T				T	C	Yes
*CytB*	14766	C to T	Thr to Ile	T/I/S/S	T	T	C	Yes
	15043	G to A			A	A	G	Yes
	15301	G to A			A	A	G	Yes
	15326	G to A	Thr to Ala	T/M/I/I	A	A	G	Yes
	15784	T to C			C		T	Yes
	15884	G to A	Ala to Thr	A/−/−/−		A	G	Yes

Abbreviation: rCRS, revised Cambridge reference sequence.

^a^Conservation assessments in human (H), bovine (B), mouse (M), and *Xenopus laevis* (X).

^b^Please see online MITOMAP (https://www.mitomap.org/MITOMAP), mtDB (https://www.genpat.uu.se/mtDB), and mtSNP (http://mtsnp.tmig.or.jp/) databases.

As shown in Figure 3C, the m.4263A > G mutation was located at the 5 ^′^‐end of the tRNA^Ile^ gene. Notably, this position was also the processing site for the tRNA^Ile^ 5 ^′^‐end precursor, which was catalyzed by RNase P and critical for tRNA identity [41, 42]. In addition, the m.4263A > G mutation disrupted a very conserved Watson–Crick base pairing (1A–69T), whereas the m.12234A > G mutation occurred at the anticodon stem of tRNA^Ser(AGY)^, a position that was very critical for tRNA structure and function. Intriguingly, the m.15927G > A mutation located at the same position of tRNA^Thr^ had been implicated in altering mitochondrial functions, apoptosis, and angiogenesis, which was involved in coronary heart disease [18]. Indeed, Position 42 was very important for proper helical conformation during genetic code decoding [43]. Therefore, it was speculated that the m.12234A > G mutation, which was similar to the m.15927G > A mutation, may also cause mitochondrial dysfunctions that were responsible for HCM. In addition, the m.4263A > G and m.12234A > G mutations were absent in 589 controls, suggesting that they may be the molecular basis for HCM in these pedigrees.

### 3.4. Increase in Plasma 8‐OHdG Levels

**Figure 4:**
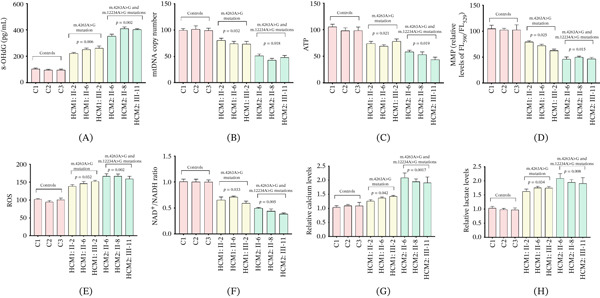
Analysis of mitochondrial functions in three patients with both m.4263A > G and m.12234A > G mutations, three subjects carrying only the m.4263A > G mutation, and three controls lacking these mtDNA mutations. (A) Analysis of 8‐OHdG concentrations in plasma. (B) Measurement of mtDNA copy number in peripheral blood. (C–H) Data from cybrid cell lines derived from patients and controls.

The presence of 8‐OHdG, induced by ROS, is widely recognized as a biomarker for oxidative stress [44]. As shown in Figure 4A, the plasma levels of 8‐OHdG in patients with both m.4263A > G and m.12234A > G mutations and subjects with only the m.4263A > G mutation were significantly higher than controls without these mtDNA mutations (*p* = 0.002 and *p* = 0.006, respectively).

### 3.5. Reduction in mtDNA Copy Number

mtDNA copy number is a measure of the number of mitochondrial genomes per cell, which is critical for mitochondrial health. Alterations in mtDNA copy number have been found to be associated with cardiovascular diseases [45]. As shown in Figure 4B, the levels of mtDNA copy number in mutant patients with only the m.4263A > G mutation and both m.4263A > G and m.12234A > G mutations were 76.5% and 48.1% of the mean values measured in control individuals (*p* = 0.032 and *p* = 0.018, respectively).

### 3.6. The Establishment of Cybrid Cell Models

To further analyze mitochondrial functions, the platelets derived from three patients with both m.4263A > G and m.12234A > G mutations (HCM2: II‐6, II‐8, and III‐11), three patients with only m.4263A > G mutation (HCM1: II‐2, II‐6, and III‐2), and three controls lacking these mutations (C1, C2, and C3) were fused into 143B *ρ* ^0^ cells. We further used direct Sanger sequencing to confirm the presence of m.4263A > G and m.12234A > G mutations in cybrids, suggesting the successful establishment of cybrid cell models.

### 3.7. Reductions in ATP Production

To explore the capacity of OXPHOS, we examined the production of ATP in nine cybrid cells. As shown in Figure 4C, the levels of ATP in the mutant cybrids carrying only the m.4263A > G mutation and both m.4263A > G and m.12234A > G mutations were 73.6% and 51.2% of the mean values analyzed in control cybrids (*p* = 0.021 and *p* = 0.019, respectively).

### 3.8. Alterations in MMP

MMP is critical for maintenance of cellular health and viability [46]. As shown in Figure 4D, the levels of MMP in cybrid cells harboring both m.4263A > G and m.12234A > G mutations and only the m.4263A > G mutation were 46.3% and 71.5% of the mean values measured in the control cell lines (*p* = 0.015 and *p* = 0.025, respectively).

### 3.9. Increase in ROS Production

ROS generated by mitochondria plays an important role in physiological role in maintaining healthy mitochondrial homeostasis [47]. As shown in Figure 4E, we found that cell lines harboring both m.4263A > G and m.12234A > G mutations and only the m.4263A > G mutation were 143.2% and 161.7% of the mean value analyzed in control cells (*p* = 0.002 and *p* = 0.032, respectively).

### 3.10. NAD^+^/NADH Ratio Analyses

The NAD^+^/NADH ratio was critical for many metabolic reactions, such as nucleotide synthesis, lipid, central carbon, and amino acid metabolisms [48]. We next examined this ratio in nine cybrid cell lines, as shown in Figure 4F. The NAD^+^/NADH ratio in cells with both m.4263A > G and m.12234A > G mutations and only the m.4263A > G mutation was 42.8% (*p* = 0.005) and 63.3% (*p* = 0.033) of the mean value measured in control cells.

### 3.11. Overload of Ca^2+^ in Mutant Cybrids

Ca^2+^ is not only important for cardiac functions but also critical for signal processing. In particular, calcium homeostasis plays an important role in cardiovascular disease pathogenesis [49]. As shown in Figure 4G, compared with control cells, significantly increased levels of Ca^2+^ were observed in cells with both m.4263A > G and m.12234A > G mutations, as well as cells with only the m.4263A > G mutation (*p* = 0.0017 and *p* = 0.042, respectively).

### 3.12. Increase in Extracellular Lactate Production

Lactate is a byproduct of glucose metabolism. Furthermore, its main production depended on glycolysis [50]. As shown in Figure 4H, the relative levels of lactate in cells carrying both m.4263A > G and m.12234A > G mutations and only the m.4263A > G mutation were 195.2% and 171.4% of the mean values measured in control cells (*p* = 0.008 and *p* = 0.034, respectively).

### 3.13. The m.12234A > G Mutation Blocked mt‐RNA Transcripts

**Figure 5:**
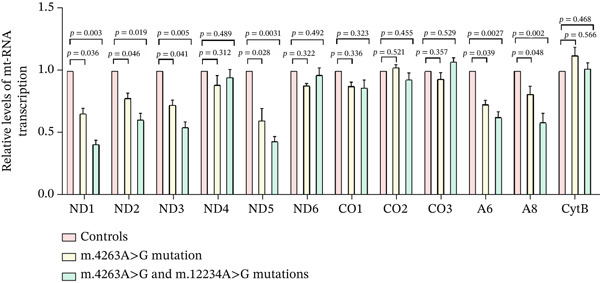
Analysis of mt‐RNA expression levels in three cybrids with both m.4263A > G and m.12234A > G mutations, three cybrids carrying only the m.4263A > G mutation, and three cybrids lacking these mtDNA mutations.

To explore whether the m.12234A > G mutation affected m.4263A > G‐induced mitochondrial dysfunctions, we analyzed the relative mt‐RNA transcription in nine cybrid cells using qPCR. As shown in Figure 5, significantly lower levels of MT‐ND1, ND2, ND3, ND5, A6, and A8 mRNA were present in cells carrying both m.4263A > G and m.12234A > G mutations when compared with cells with only m.4263A > G and control cells lacking these mtDNA mutations. This result indicated that the m.12234A > G mutation might, at least, partially block mt‐RNA transcription.

## 4. Discussion

HCM is often hereditary, and thus, family screening is generally recommended to identify relatives at risk of disease development. Although this disease has been reported to be associated with many variants of genes involved in sarcomeric protein biomechanics, pathogenic genes have not yet been identified in patients with HCM. Previous studies demonstrated that most known HCM genetic variants lead to higher myocardial energy demand [51]. The imbalance between inefficient energy supply and increased ATP demand leads to adverse remodeling and contributes to the progression of this disease [52]. Mitochondria play a central role in cardiac energy metabolism. mtDNA mutation–associated mitochondrial dysfunctions have been described in patients and animal models of HCM [53, 54]. As adapter molecules to convert the genetic information stored into amino acid sequences, mt‐tRNA plays a critical role in protein synthesis. Although mt‐tRNA only accounted for 10% of the total coding capacity of mitochondrial genomes, more than one‐half of mitochondrial disease‐related mtDNA mutations were located in this region, highlighting the importance of mt‐tRNA mutations to mitochondrial functions [55].

Herein, we reported the clinical, genetic, and biochemical features of two Chinese pedigrees with HCM. Interestingly, HCM as a sole clinical phenotype was only found in matrilineal relatives of two families, strongly indicating that mitochondrial dysfunctions were the important molecular basis for this disease. Clinical evaluations revealed that the penetrances and expressivities of HCM2 were much higher than those of the HCM1 pedigree, despite the fact that these patients exhibited different ages at onset of HCM. Thus, identification of HCM‐causative genes was of paramount importance for investigating its complex mechanism, as well as its therapeutic targets.

Considering HCM was mainly attributed to mutations in nuclear genes, in particular, the sarcomeric genes such as *MYH7*, *MYBPC*, and *TNNI3* accounted for more than 80% of all HCM cases [56]. We, therefore, first performed WES to screen the nuclear gene mutations. Unfortunately, our data indicated that no loss‐of‐function mutations were identified, suggesting that nuclear genes may not play active roles in HCM pathogenesis.

Mutational analysis of mitochondrial genomes from HCM1 and HCM2 families revealed the occurrence of 43 mutations (1 novel and 42 known). These genetic mutations were further evaluated for evolutionary conservation, lower frequency in control subjects, and potential structural and functional alterations of the corresponding genes. As a result, we identified loss‐of‐function mutations in mt‐tRNA^Ile^ and mt‐tRNA^Ser(AGY)^ genes. The occurrence of the m.4263A > G and m.12234A > G mutations in two genetically unrelated pedigrees affected by HCM and differing considerably in their mtDNA haplotypes (D4j and D4), as well as their absence in 589 controls, strongly indicated that these mt‐tRNA mutations were involved in the pathogenesis of HCM. In fact, the m.4263A > G mutation was localized at the processing site for the tRNA^Ile^ 5 ^′^‐end precursor, which was catalyzed by RNase P [57]. Notably, this mutation also changed the stop codon TAA of the *ND1* mRNA to an equivalent TAG stop codon and disrupted a conserved base pairing (1A–69T). Thus, it was hypothesized that the m.4263A > G mutation impaired tRNA 5 ^′^‐end processing and its metabolism. According to a previous study [58], cybrid cells carrying the m.4263A > G mutation exhibited an approximately 46% reduction in the steady‐state level of tRNA^Ile^, as assessed by Northern blot analysis. Furthermore, the m.4263A > G mutation reduced mitochondrial protein synthesis, affected OXPHOS capacity, and led to mitochondrial dysfunctions [58]. Therefore, the m.4263A > G was a definitely pathogenic mutation for HCM.

Cardiomyopathy‐associated pathogenic mutations were frequently distributed across the mt‐tRNA genes, with more than one‐third residing in just three of these genes: tRNA^Leu(UUR)^, tRNA^Ile^, and tRNA^Lys^ [59, 60]. By contrast, the tRNA^Ser(AGY)^ seemed to be the least affected mt‐tRNA gene. The A‐to‐G substitution at Position 12234 resided at Position 42 in the anticodon stem of tRNA^Ser(AGY)^, as the anticodon stem played crucial roles in decoding the mRNA surrounded by other nucleotides that impacted its efficiency and binding to mitochondrial seryl‐tRNA synthetase (mt‐SerRS). Thus, mutations in the anticodon stem may be more sensitive to reducing its biological activity, especially for the noncanonical mt‐tRNA^Ser(AGY)^, which lacked the entire D‐loop and D‐stem structures [61]. Interestingly, nucleotides at the same position of tRNA^Ile^ (m.4300A > G) and tRNA^Thr^ (m.15927G > A) were regarded as pathogenic mutations for mitochondrial cardiomyopathy [62, 63]. Thus, it can be speculated that the m.12234A > G mutation may be similar to the m.4300A > G or m.15927G > A mutation, leading to a failure in tRNA metabolism and causing mitochondrial dysfunctions that were involved in HCM.

The altered tRNA^Ile^ and tRNA^Ser(AGY)^ metabolisms caused by the m.4263A > G and m.12234A > G mutations led to a defect in mt‐RNA transcription. In particular, the relative mRNA levels of ND1, ND2, ND3, ND5, A6, and A8 were significantly lower in cybrids with both tRNA^Ile^ and tRNA^Ser(AGY)^ mutations than in cybrids with only the tRNA^Ile^ mutation, as well as control cells without these mutations. This result suggested that defects in mt‐RNA translation caused by both m.4263A > G and m.12234A > G mutations seemed to be more responsible for multiple OXPHOS complex deficiencies in cybrids. Mitochondrial functional analyses revealed that patients with both mutations had a significantly lower mtDNA copy number in peripheral blood. Furthermore, cybrid cell lines harboring both mutations exhibited much lower levels of ATP production, MMP, and NAD^+^/NADH redox ratio compared with control cybrids. By contrast, the relative levels of 8‐OHdG, ROS, Ca^2+^, and lactate were dramatically increased. These data indicated that the combination of the m.4263A > G and m.12234A > G mutations resulted in more severe mitochondrial dysfunctions than individuals with only the m.4263A > G mutation and controls without these mtDNA mutations. In particular, the mtDNA copy number, which stands for the number of mtDNA in each cell, is believed to be an indicator of mitochondrial function, and reductions of mtDNA copy number were closely related to cardiovascular diseases [64]. In addition, electron transport chain (ETC) defects will subsequently lead to a decrease in MMP, which resulted from redox transformations associated with the Krebs cycle and served as a form of energy storage [65]. Because ATP production and MMP required the universal cofactor NAD^+^ as an essential enzyme, NAD gained two electrons and a proton from substrates in the Krebs cycle, being reduced to NADH [66]. Thus, the optimal NAD^+^/NADH redox ratio was needed for efficient mitochondrial metabolism [67]. A marked decrease in this ratio and increase in ROS and 8‐OHdG concentrations were observed in patients from the HCM2 pedigree, indicating that the combination of the m.4263A > G and m.12234A > G mutations increased oxidative stress and caused imbalance of redox status, as in the case of deafness‐associated m.1555A > G and m.4317A > G mutations [68]. Furthermore, Ca^2+^ played a fundamental role in the regulation of electrophysiological signaling in cardiomyocytes [69]. Excessive Ca^2+^ concentration within the mitochondrial matrix elevated mitochondrial ROS production and led to the activation of proapoptotic factors, eventually causing cardiomyocyte death [70]. Lactate was implicated in many biological processes, including energy regulation, fatty acid metabolism, and controlling the redox balance [71]. Lactate has also been used as an indicator for disease diagnosis, prognosis, and efficacy evaluation in clinical settings [72]. Thus, the m.4263A > G and m.12234A > G mutations led to an increase in lactate levels, implying that abnormal energy metabolism was involved in HCM pathogenesis.

## 5. Conclusions

In summary, our study demonstrated the role of a novel HCM‐related allele (m.12234A > G mutation) in the tRNA^Ser(AGY)^ gene in the clinical expression of HCM‐associated tRNA^Ile^ 4263A > G mutation. The m.12234A > G mutation altered the structure and function of tRNA^Ser(AGY)^ and aggravated mitochondrial dysfunctions, enhancing the penetrance of HCM caused by the m.4263A > G mutation. Thus, our investigation provided novel insight into the pathophysiology of HCM that was caused by mt‐tRNA mutations.

## Author Contributions

Yu Ding and Daojun Yu designed the study and wrote the manuscript. Mengting Liu and Shunrong Zhang collected samples and performed molecular, genetic, and statistical analyses. Qinxian Guo performed cellular experiments. All authors have read, corrected, and made significant contributions to this manuscript.

## Funding

This study was funded by the Key Project of Natural Science Foundation of Zhejiang Province, LZ22H190002; the Hangzhou Joint Fund of the Zhejiang Provincial Natural Science Foundation of China, LHZY24H020002; the Key Project of Hangzhou Bureau of Science and Technology, 202204A01; the Zhejiang Provincial Medicine and Health Science Foundation, WKJ‐ZJ‐2514; and the Construction Fund of Key Medical Disciplines of Hangzhou, Laboratory Diagnostics, 2025HZZD01.

## Conflicts of Interest

The authors declare no conflicts of interest.

## Data Availability

The raw data supporting the conclusions of this article will be made available by the authors upon request (dingyu_zj@126.com).
